# Rotenone Exposure During Development Conditions Parkinsonian Phenotype in Young Adult Rats

**DOI:** 10.3390/toxics13040290

**Published:** 2025-04-10

**Authors:** Margarita Gómez-Chavarín, Patricia Padilla, Mireya Velázquez-Paniagua

**Affiliations:** 1 Physiology Department, Medicine School, National University of Mexico, Ciudad de México 04500, Mexico; yetlanetzi@unam.mx; 2 Liquid Chromatography Unit, Biomedical Research Institute, National University of Mexico, Ciudad de México 04500, Mexico; ppadillac@biomedicas.unam.mx

**Keywords:** dopaminergic neurons, *Substantia nigra* (*S. nigra*), *Caudate nucleus* (*C. nucleus*), rotenone (ROT), tyrosine hydroxylase (TH), α-synuclein in soluble form (α-syn_if_), global DNA methylation (5-mC DNA)

## Abstract

Current studies suggest that environmental toxins may play a significant role in the fetal origins of Parkinson’s disease (PD). Significant evidence from animal experiments has demonstrated that these toxins can disrupt fetal neurodevelopment. PD is a neurodegenerative disorder related to the loss of dopaminergic neurons in the substantia nigra pars compacta (*S. nigra*) and accumulation of α-synuclein (α-syn) in the brain. Parkinson’s disease has long been associated with an idiopathic etiology, with environmental or ontogenetic factors as causes; however, the list of causal agents continues to expand as their effects are investigated at different stages of development. To explore the potential ontogenetic origins of PD, we exposed female rats subcutaneously (s.c.) to 1 mg/kg of the pesticide rotenone (ROT)—21 days during gestation, 21 days of breastfeeding, or 42 days in both periods—and assessed its long-term effects on their pups in adulthood. Our findings reveal that ROT exposure induces the degeneration of dopaminergic neurons in the *S. nigra* of adult rats. We administered ROT to dams during specific developmental stages and examined the nigrostriatal pathway and its functionality in offspring upon reaching young adulthood. Our results showed that perinatal ROT exposure led to (1) diminished motor skills, (2) greater concentrations of α-syn in the caudate nucleus (*C. nucleus*) and *S. nigra*, (3) reduced numbers of tyrosine hydroxylase immunoreactive neurons, and (4) hypomethylation of global 5-methylcytosine DNA compared to control rats at 60 days of age. The effects were more pronounced in rats exposed to ROT in utero and in both the in utero and breastfeeding periods, with fewer effects observed in those exposed only during breastfeeding. Thus, our findings suggest that exposure to ROT during the early developmental stages predisposes rats to Parkinsonian symptoms later in adulthood.

## 1. Introduction

Parkinson’s disease (PD) is a progressive neurodegenerative disorder characterized by a range of symptoms including autonomic, motor, cognitive, and psychiatric disorders. It is marked by the loss of dopaminergic neurons in the substantia nigra (*S. nigra*) and parkin, which are associated with a lack of degradation of ubiquitinated proteins and accumulation of α-synuclein in Lewy bodies and parkin [1,2,3,4,5]. Although a certain proportion of PD cases involve a genetic etiology [6,7,8]; recent epidemiological and experimental evidence suggests that the etiology of adult sporadic PD, the most common form of the disease, may involve environmental influences and is associated with sex [9,10,11]. Indeed, exposure of adult animals, including humans, to heavy metals, pesticides, herbicides, and other environmental pollutants and neurotoxins has been shown to induce Parkinsonian signs and symptoms later in life [12].

Moreover, environmental factors might not only induce Parkinsonian-like symptoms after adult exposure but also during prenatal and/or early postnatal development [13].

Evidence suggests that exposure to pesticides [12,13,14,15,16,17], heavy metals [18,19,20], cocaine [21], infections [22,23], inflammation [24], and/or even a mother’s high caloric intake [25,26] during these critical periods increases the risk of developing adult Parkinsonism.

For instance, adult rats chronically exposed to rotenone (ROT), a natural pesticide used in agriculture, develop motor dysfunctions and show reduced numbers of nigral dopaminergic neurons and increased availability of the insoluble form of α-syn protein (α-syn_if_) in the caudate nucleus (*C. nucleus*) and *S. nigra* [3,13,27,28,29,30,31].

Rotenone (ROT), a pesticide derived from some plant species, exerts neurotoxicity upon inhalation, ingestion, and exposure via dermal penetration and absorption [32]. This is accomplished by inhibiting complex I of the electron transport chain in mitochondria [33], causing mitochondrial toxicity and oxidative stress, decreasing microtubule polymerization, thus inducing a shortening of neurite length in both dopaminergic and non-dopaminergic neurons [34] (McKnight and Hack, 2020). Rat models have demonstrated that ROT exposure can induce PD symptoms such as bradykinesia, postural instability, and rigidity, with epidemiological studies showing higher rates of PD in individuals with ROT exposure than in their unexposed counterparts [34]. Given the ongoing controversy surrounding the ontogenetic origins of adult PD, in this study, we aimed to investigate whether dams exposed to ROT during gestation and/or breastfeeding, critical periods for the early development of dopaminergic neurons in their progeny, predispose their offspring to develop parkinsonian signs in adulthood. Additionally, we sought to determine whether rats exposed to ROT in utero exhibited more severe neurological deficits than those exposed during breastfeeding, or whether exposure during both periods produced additive effects. Thus, our study sought to elucidate the influence of maternal ROT exposure during the neurodevelopmental stages of their progeny on the manifestation of Parkinsonian symptoms in adulthood.

## 2. Materials and Methods

### 2.1. Dams Breeding

Wistar rats from our breeding colony, aged eight weeks and weighing 250–280 g, were used in this study. The animals were housed in standard cages under controlled environmental conditions, including a 12:12-h light–dark cycle and a temperature maintained between 20 and 22 °C, with ad libitum access to food and water provided. Primiparous females were paired with one male breeder and housed twice per cage. Pregnancy was confirmed on day 1 by vaginal smear analysis. Pregnant females were randomly assigned to either the control (Ctrl) group or the group treated with ROT during gestation and breastfeeding. A rotenone (ROT; R8875, Sigma-Aldrich, St. Louis, MO, USA) dose of 1 mg/kg/day in 100 μL of polyethylene glycol 200 (25322-68-3, J. T. Baker, Phillipsburg, NJ, USA) and the route of administration were selected based on previous studies indicating that this dose ensures the survival of both offspring and dams [13]. ROT was administered via subcutaneous (s.c.) injections to dams daily for approximately six weeks. Different groups were formed to analyze ROT exposure at different developmental stages, as described below, from 3 weeks to 21 days of age during breastfeeding until weaning. Animal handling and experimental procedures were carried out in accordance with the rules established by the Mexican Official Standard Norm [35] and the guidelines outlined in the Guide for the Care and Use of Laboratory Animals published by the Medicine School of the National University of Mexico, and you can find it at https://di.facmed.unam.mx/comisiones/Acta_cicual.pdf, accessed on 1 January 2020. None of the animals were euthanized for humane reasons. The protocol for animal care and welfare was carried out by MVZ Ismael Torres from the Bioterium Unit of the Medicine School of the UNAM (Supplementary Materials S1).

### 2.2. Pups and Cross-Fostering

**Figure 1:**
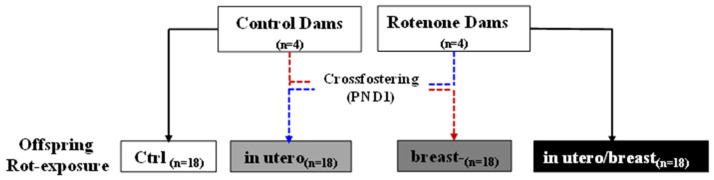
Scheme of offspring ROT-exposure during different neurodevelopmental stages and cross-fostering at 1 PND (postnatal day).

On the postnatal day (PND), 72 pups were divided into distinct groups as follows: control (Ctrl; *n* = 36) and ROT 1 mg/kg (ROT; *n* = 36). Pups were individually marked with tail identification and assigned to different groups: control (Ctrl; 10 males and 8 females), those treated with ROT exclusively during breastfeeding (breast; 9 males and 9 females), those exposed to ROT solely in utero (in utero; 8 males and 10 females), and those exposed to ROT both in utero and during breastfeeding (in utero/breast; 11 males and 7 females). (see Supplementary Table S2). To ensure a balanced distribution, cross-fostering was implemented by exchanging half of the litter between dams (Figure 1). Ctrl pups were born to dams that received no treatment, and offspring born to mothers treated with ROT from the first day of gestation (in utero) were nursed after birth by control dams that did not receive ROT while breastfeeding. Pups exposed to ROT during breastfeeding (breast, 21 days until weaning) were born from Ctrl dams, but nursed after birth by ROT-treated lactating dams. ROT-exposed pups in utero/breastfeed (in utero/breast) were born to dams treated with ROT at both stages (42 days). All pups were weaned at 21 days of age and weighed on days 1, 30, and 60 after birth, regardless of the treatment exposure. Animal handling and experimental procedures were carried out in accordance with the rules established by the Mexican Official Standard Norm (35) and the guidelines outlined in the Guide for the Care and Use of Laboratory Animals published by the Medicine School of the National University of Mexico and available at https://di.facmed.unam.mx/comisiones/Acta_cicual.pdf. None of the animals were euthanized for humane reasons. The protocol for the care of animal welfare was carried out by MVZ Ismael Torres from the Bioterium Unit of the Medicine School of the UNAM (Supplementary Materials S2).

### 2.3. Motor Coordination Assessment

To assess the motor performance of the adult offspring from each group, we used the motor coordination beam test established by Drucker-Colín and García-Hernandez [36]. All young adult offspring rats underwent a five-day training regimen between 9:00 am and 11:00 am. During this period, they were familiarized with traversing a beam measuring two meters in length and 24 mm in width, inclined at an angle of 15°. Rats were placed at the lower end of the beam, and the time taken to reach the home cage located at the far end was recorded (measured as latency in seconds). On the sixth day, the testing phase was initiated within the same environment and timeframe (9:00 and 11:00). During testing, the width of the beam was changed randomly across consecutive trials, with widths of 9, 6, or 3 mm utilized interchangeably, based on a predetermined contingency table. The latency time (seconds) required for each rat to complete the test was recorded. When a rat exceeded the predefined latency time (120 s) without successfully crossing the beam, it was removed by hand and returned to its home cage, with a score of 120 s assigned for that trial.

### 2.4. Quantification of Insoluble Forms of α-Syn (α-Syn_if_)

To quantify the content of α-syn_if_ in the *C. nucleus* and *S. nigra* of young adult offspring, rats from each group previously assessed for motor coordination were euthanized by decapitation under deep anesthesia with sodium pentobarbital (18.5 mg/kg i.p.; Pfizer, Mexico City, Mexico). Their brains were swiftly removed, and tissue samples were processed as described by Campbell et al. [37]. The concentration of α-syn_if_ was estimated using the Biotrak ELISA System, as suggested by the supplier (RPN 5902; Amersham Biosciences, Buckinghamshire, UK). ELISA assays were performed in triplicate. The intra-assay coefficient of variation was <5%. We generated a standard curve using linear regression (α-synuclein-insoluble form (pg/mL); Supplementary Figure S1).

### 2.5. Estimation of ROT Concentration in Blood Serum in Pups

For the identification of ROT in blood samples, 200 µL of each sample was collected from 6 pups per experimental condition. These samples were centrifuged at 3000 rpm for 10 min at room temperature to separate the plasma, which was then stored at −70 °C for subsequent instrumental analysis. Prior to chromatographic analysis (HPLC), the plasma was precipitated with acetonitrile (*v*/*v*) and the resulting supernatant was extracted via centrifugation and vacuum evaporation. The precipitate was reconstituted with 30 µL of HPLC-grade acetonitrile and 10 µL was injected into the system. A Waters HPLC-UV system equipped with a SunFire C18 column (5 μm diameter, 4.6 mm × 150 mm, (cat. 186002559; Milford, MA, USA) was used to separate some blood serum components. The mobile phase consisted of a gradient of H_2_O and 100% acetonitrile. The gradient started with a 10% aqueous solution (A) and 90% acetonitrile (B), shifted to 70% A and 30% B after 2 min, and reached 75% A and 25% B after 12 min. The flow rate was maintained at 1.0 mL/min at room temperature. The compound of interest, ROT, was detected by measuring UV absorbance at a wavelength of 290 nm. Detection and quantification of ROT were performed using Empowered^®^ software (Version 71500031203; Milford, MA, USA). The retention time of ROT was determined to be 7.35 min. The developed method exhibited sensitivity, with a limit of detection (LOD) of 0.12 µg/mL and limit of quantification (LOQ) of 0.35 µg/mL. The method demonstrated linearity (r^2^ > 0.999) within a quantification range of 0.25 to 250 µg/mL), accuracy (94 < %R < 112), selectivity (no endogenous or exogenous interferences were found), and reproducibility (CV < 7.81%).

### 2.6. Tyrosine Hydroxylase Immunocytochemistry

Young adult offsprings rats (60 days of age) from each experimental group underwent deep anesthesia induced by sodium pentobarbital (18.5 mg/kg i.p.; Pfizer, Cd. Mx.Mexico) and were transcardially perfused with 250 mL of phosphate-buffered saline (PBS; 0.1 M, pH 7.4) followed by 250 mL of buffered paraformaldehyde (4%). Subsequently, the brains were removed and post-fixed overnight in the same fixative solution, cryoprotected using graded sucrose solutions (15 and 30%) at 4 °C. Serial coronal slices (40 μm thick) spanning the entire *S. nigra* were obtained using a freezing microtome (Cryo-Cut American Optical, ON, Canada) and collected into 24-well culture dishes filled with PBS. Immunoperoxidase staining for tyrosine hydroxylase (TH) was performed according to established protocols. Primary rabbit polyclonal antibodies against TH (sc-14007; Santa Cruz Biotechnology, Dallas, TX, USA) were diluted 1:1000 in PBS supplemented with 0.3% Triton X-100, followed by incubation with anti-rabbit IgG polyclonal biotinylated secondary antibodies made in goat diluted 1:200 in PBS-triton (BA-1000; Vector Laboratories). The sections were treated with avidin-biotin peroxidase according to the manufacturer’s instructions (Standard Elite kit, Cat. PK-6100; Vector Laboratories, Burlingame, CA, USA) for 2 h at room temperature, washed with PBS, and incubated with a solution containing 2,2-diaminobenzidine (DAB) and hydrogen peroxide as per the supplier’s recommendations (peroxidase substrate kit DAB; Cat. No. SK-4100; Vector Laboratories, Burlingame, CA, USA). The slices were mounted onto gelatin-subbed slides and coverslipped using Cytoseal 60 (Cat. No. 8310-4; Thermo Fisher Scientific, Waltham, MA, USA).

### 2.7. Quantification of Dopaminergic Neurons in the S. nigra

TH-immunostained sections of *S. nigra* from each experimental group were imaged and digitized using a CCD color video camera, Reichert–Jung interface (9009B0; Depew, NY, USA) and Matrox PC-VCR (IAI-CV-S3200, Jay Corp., Tokyo, Japan), controlled by a computer-based image analysis system (Image-Pro Plus 11.1.1, MD, USA). Consistent brightness, gain, and contrast settings were maintained throughout the image-acquisition sessions. Single fields of TH-stained *S. nigra* (40× magnification) were randomly selected from each section that contained the neuronal nucleus. Twenty sections of the right side of the brainstem were photographed. TH-immunoreactive (TH-IR) neurons were manually counted over an area of 0.075 µm^2^ per section. Only TH-IR neurons with soma diameters ranging from 12 µm to 20 µm and clearly visible cell nuclei were included in the counting process [38]. Notably, the individuals conducting neuron counting were blinded to the experimental conditions.

### 2.8. Global DNA Methylation (5-mC) in S. nigra

DNA extraction was performed using a Qiagen DNA Mini Kit, according to the manufacturer’s instructions (cat. 56304; Qiagen, Hilden, Germany) in rats exposed to ROT during neurodevelopment and control groups. The quality and concentration of the DNA were assessed at 10 ng/μL using a NanoDrop Spectrophotometer (NS-1000, Thermo-Fisher, Waltham, MA, USA). Only weqccDNA samples with 260/280 ratios close to 1.8 were included in the study.

To measure the global 5-mC DNA levels in the *S. nigra* of adult offspring exposed to ROT and the control group, the brain tissue was dissected under a microscope and stored at 70 °C for subsequent analysis using an ELISA-like colorimetric methylated DNA quantification kit (P1030-96; Epigentek, New York, NY, USA), following the manufacturer’s instructions. A total of 100 ng of DNA was used, and the absorbance was measured at 450 nm using a Microplate Reader (BioTek Instruments, Friedrichshall, Germany).

To quantify the absolute amount of methylated DNA, we generated a standard curve using linear regression (5-mC standard (%)] (Supplementary Figure S2). The amount and percentage of 5-mC were calculated using the following formulae:

5-mC (ng) = (Sample OD − Blank OD)/(Slope × 2)(1)

5-mC (%) = 5 mC (ng)/sample DNA (ng) × 100(2)

### 2.9. Statistical Analysis

The results were presented as mean values of the measured parameters and their standard errors (SEM). The body weights of dams and offspring were assessed using a two-way ANOVA. Differences in TH-IR neurons, concentration of ROT in the blood, latency, and α-syn_if_, as well as multiple range comparisons between the groups, were assessed using the Kruskal–Wallis test with Dunn’s multiple comparisons. Meanwhile, differences in %5-mC DNA were assessed using ordinary ANOVA and Tukey’s multiple comparisons. Statistical significance was set at *p* < 0.05.

Furthermore, the relationship between the number of TH-IR neurons and α–syn_if_ concentration in the *S. nigra*, as well as the values of beam climbing latency (s) and TH-IR in *S. nigra,* and α–syn_if_ concentration in *C. nucleus* and *S. nigra* among the different groups were compared using one-tailed Pearson’s simple linear correlation tests. All statistical analyses and graphs were generated using Prism version 10.1.1. (GraphPad Software, Inc., La Jolla, CA, USA).

## 3. Results

### 3.1. Body Weight Gain in Dams and Pups

**Figure 2:**
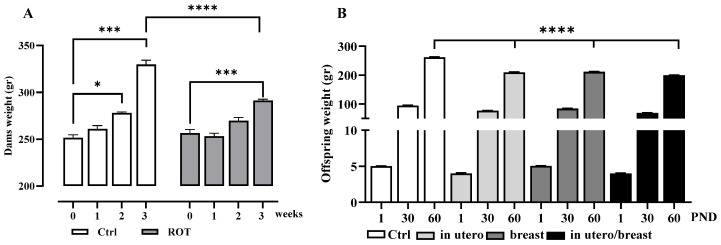
Body weight of pregnant dams and offsprings. Body weight (g) of dams (**A**) recorded at 1, 2, and 3 weeks of pregnancy in Ctrl and following treatment with ROT (*n* = 8) and (**B**) body weight in all offspring groups at birth, 30 and 60 DPN (*n* = 72).

To evaluate the impact of rotenone exposure on different developmental stages, the pups were divided into four groups, as depicted in Figure 1. The Integrated Risk Information System of the USA established the Lowest Observed Effect Level (LOEL) for ROT at 6 mg/kg/day to induce fetotoxic effects in pregnant rats, resulting in diminished body weight in both dams and offspring. In our study, we opted for a minimal dose of 1 mg/kg/7 weeks over an extended period to ensure maximum survival of both dams and offspring. Our decision was based on prior observations indicating that ROT doses of 2 mg/kg/7 weeks decrease offspring survival and induce an inability in dams to give birth (unpublished results). Intrauterine growth, a key indicator of fetal well-being, is significantly affected by failure, resulting in underweight offspring at birth. Figure 2A illustrates the differences in body weight gain in dams induced by ROT exposure during gestation at 1, 2, and 3 weeks, highlighting the notable weight loss during the third week of gestation following treatment with ROT, and in offspring (Figure 2B), no disparity in body weight at birth, and no weight gain at 30 DPN, but differences emerged at 60 PND. The results represent the mean ± SEM. Asterisks indicate values from the two-way ANOVA (* *p* < 0.05, *** *p* < 0.001, **** *p* < 0.0001).

### 3.2. ROT Exposure In Utero and/or Breast-Impairs Motor Coordination

The motor coordination test utilized in this study serves as a reliable quantitative measure of motor capability disturbances in rats with nigrostriatal dysfunction [36]. Briefly, all rats underwent behavioral testing between 9:00 and 11:00 a.m. and were tasked with walking and climbing a beam tilted at an angle of 15°. The test was conducted under consistent environmental conditions, with the beam width randomly varying between 9, 6, or 3 mm in consecutive trials using a contingency table. Latency time, which indicates the amount of time each rat took to complete the test, was recorded. Rats that failed to cross the beam within the allocated time were manually removed, returned to their home cage, and received a score of 120 s.

**Figure 3:**
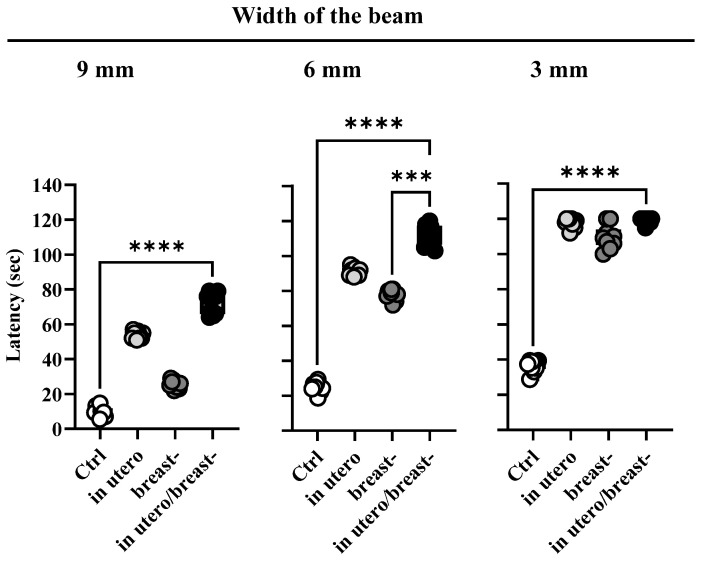
Motor impairment induced by rotenone exposure. Latency (s) for traversing the beam-climbing of 9, 6, and 3 mm width and 15° tilted beam of Ctrl and ROT-exposed rats in utero, breast- or both (*n* = 10 for each group). *** *p* < 0.005; **** *p* < 0.0001.

Pregnant and breastfeeding dams were subjected to ROT exposure to evaluate their impact on the motor behavior of their adult offspring using tilted beams of three different widths. Overall, all rats exposed to ROT exhibited increased beam-climbing latency compared with the control group (Figure 3), indicating impaired motor coordination in ROT-exposed animals. This deficit was most notable in rats exposed to ROT in utero/breast, followed by those exposed during in utero or breast alone, particularly when climbing 9 mm and 6 mm wide beams. Latency time peaked similarly across all ROT-exposed rats when confronted with a 3 mm-wide beam, with no differences between females and males. Values are presented as standard deviation. A one-way ANOVA followed by Tukey’s multiple comparison test was used to reveal significant differences in the latency time required to execute the behavioral test (s), F_(11,108)_ = 1076, *p* < 0.0001, and r^2^ = 0.9910.

### 3.3. Exposure to Rotenone In Utero and/or Breastfeeding Decreases the Number of TH-IR Neurons in S. nigra and Increases the Concentration of α-Syn_if_ in S. nigra and C. nucleus

**Figure 4:**
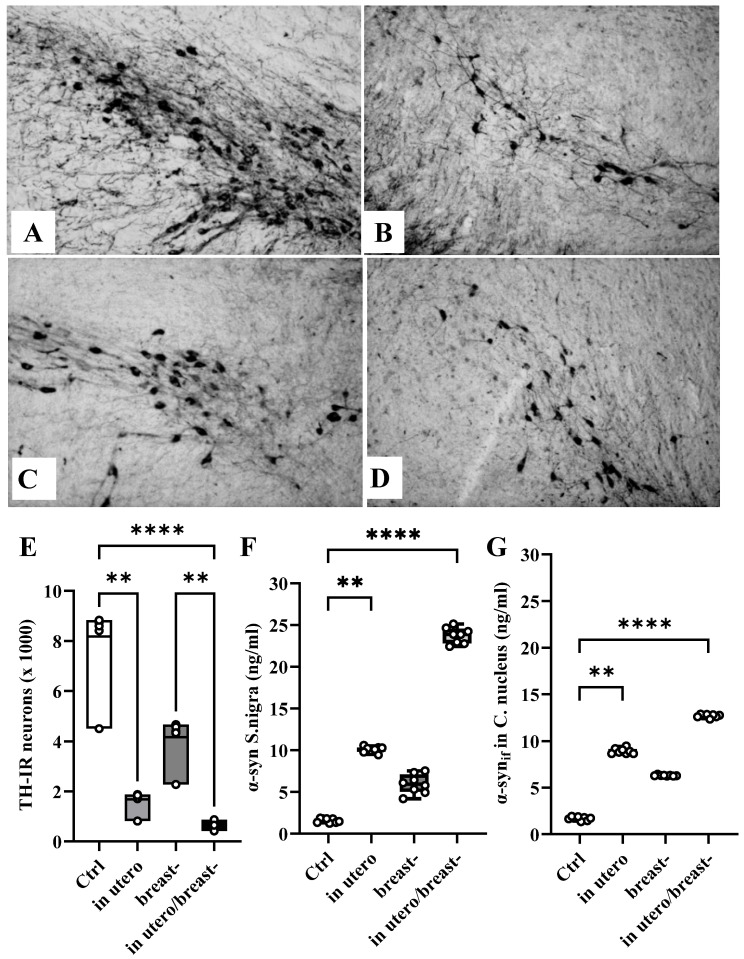
Tyrosine hydroxylase and α-syn_if_ of rotenone exposed rats. (**A**–**D**) Photomicrographs illustrate the immunocytochemical staining pattern for tyrosine hydroxylase (TH-IR) observed in the *S. nigra* of Ctrl rats (**A**), and in rats exposed to ROT during in utero (**B**), breast- (**C**) or in utero/breast- conditions (**D**). The reduction in the number of dopaminergic neurons in animals exposed to ROT can be noted qualitatively (**A**–**D**) and quantitatively (*n* = 4 for each group) (**E**). Graph (**F**,**G**) depicts the concentration of the insoluble form of α-synuclein (α-syn_if_) concentration in the *S. nigra* and *C. nucleus* of Ctrl rats compared to those exposed to ROT under the different experimental conditions. (*n* = 8 for each group) ** *p* < 0.05; **** *p* < 0.001.

Pregnant and breastfeeding dams underwent subcutaneous injection of ROT to investigate its impact on the number of dopaminergic neurons in *S. nigra*, assessed through TH-IR immunostaining in their adult descendants. Building on prior research [13], which demonstrated a dose–response relationship to 1 mg/kg ROT s.c. in pregnant and breast dams, resulting in reduced dopaminergic neuron numbers in both dams and their offspring, we opted for subcutaneous administration. This route allows for the administration of small volumes, ensuring slow and prolonged absorption with sustained effects, which is particularly advantageous considering the physiological conditions of pregnant dams. The choice for subcutaneous administration was motivated by the fact that the primary route of absorption of fat-soluble pesticides and toxic substances in humans is through the skin, a pathway often overlooked, despite the skin being the largest organ in both humans and animals (approximately 2 m^2^). We observed a reduction in the number of TH-IR neurons in the *S. nigra* of all offspring rats 60 DPN exposed to ROT at all developmental stages. Notably, this reduction was more pronounced in rats exposed to ROT in utero/breast (92%) than in those exposed solely in utero (80%) or breast (50%), in contrast to the 100% observed in the control group (Figure 4E). A Kruskal–Wallis test and Dunn’s multiple comparisons test were used to reveal significant differences (F_(4,32)_ = 28.56, *p* < 0.0001).

After confirming the deleterious effects of ROT on the number of dopaminergic neurons, we estimated the concentration of α-syn_if_ in *S. nigra* and *N. caudate* at 60 PND. In all the animals exposed to ROT, the ELISA analyses revealed increments of α-syn_if_ in the *S. nigra* (Kruskal–Wallis’ test and Dunn’s multiple comparisons test were used to reveal significant differences (F_(4,32)_ = 29.09, *p* < 0.0001; Figure 4F) and *N. caudate* (ANOVA with Kruskal–Wallis’ test and Dunn’s multiple comparisons test were used to reveal significant differences (F_(4,32)_ = 29.09, *p* < 0.0001; Figure 4G) compared with the Ctrl group. Notably, the increment was 15 times greater in animals exposed to ROT in utero/breast, followed by six times in those exposed during in utero, and four times in those exposed only during breast. The concentration of α-syn_if_ in *S. nigra* was higher than that in *N. caudate* under all experimental conditions. This suggests that the dopaminergic pathway is highly susceptible to ROT neurotoxicity when administered to both structures during neurodevelopment.

### 3.4. Concentration of ROT in Blood Serum in Pups

**Figure 5:**
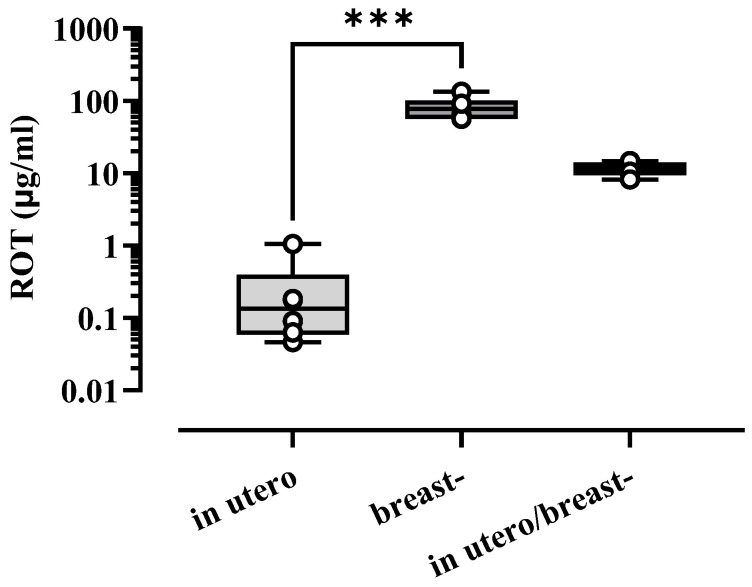
Concentration of ROT in the rat offspring. Graph illustrates the concentration of ROT (µg/mL) in the blood serum of offspring rats at 30 PND, determined by a HPLC system. No ROT was detected in offspring at 60 PND. PND: postnatal day (*n* = 6 for each group). *** *p* < 0.001.

Following exposure to ROT at any developmental stage, blood serum from adult offspring rats at 30 and 60 PND was chemically analyzed to quantify the concentration of ROT in the blood plasma. As depicted in Figure 5, the ROT concentration was higher in offspring at 30 PND during in utero/breast exposure than in breast and in utero alone, with concentrations of 0.2673, 82.41 and 11.72 µg/mL, respectively. The Kruskal–Wallis test with Dunn’s multiple comparisons test showed [F_(3,18)_ = 15.16, *p* < 0.0001]. No ROT was detected in the offspring at 60 PND.

### 3.5. Correlation Between α-Syn_if_ Content and Dopaminergic TH-IR Neuron Number in S. nigra, andα-Syn_if_ content in C. nucleus and Motor Coordination

**Figure 6:**
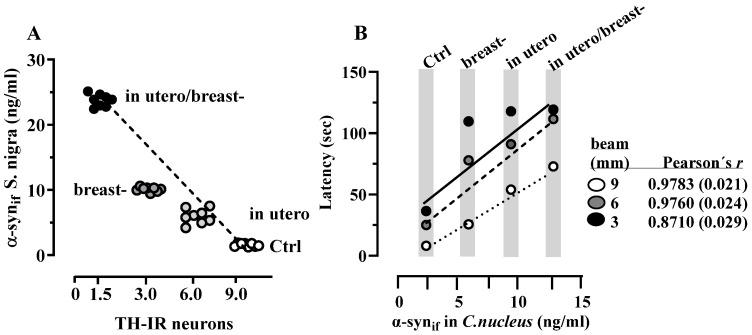
Pearson’s linear correlation plots of α-syn. The Pearson’s linear correlation plot depicts in (**A**) the negative relationship between the concentration of the insoluble form of α-syn_if_ and number of dopaminergic neurons TH-IR in the *S. nigra*; and in (**B**) the positive relationship between beam climbing latency and the concentration of α-syn_if_ in the *C. nucleus* of rats Ctrl and ROT-exposed in utero, breast, or both.

Pearson’s linear correlation tests revealed a significant negative correlation between the reduction in the number of dopaminergic neurons in the TH-IR and α-syn_if_ concentrations in *S. nigra* (r = −0.9738; *p* = 0.026; Figure 6A). Additionally, this statistical test revealed a positive correlation between the increase in α-syn_if_ in the nucleus and elevated beam-climbing latencies in the ROT-treated animals (Figure 6B).

### 3.6. α-Syn_if_ and Global 5-mC DNA in S. nigra

**Figure 7:**
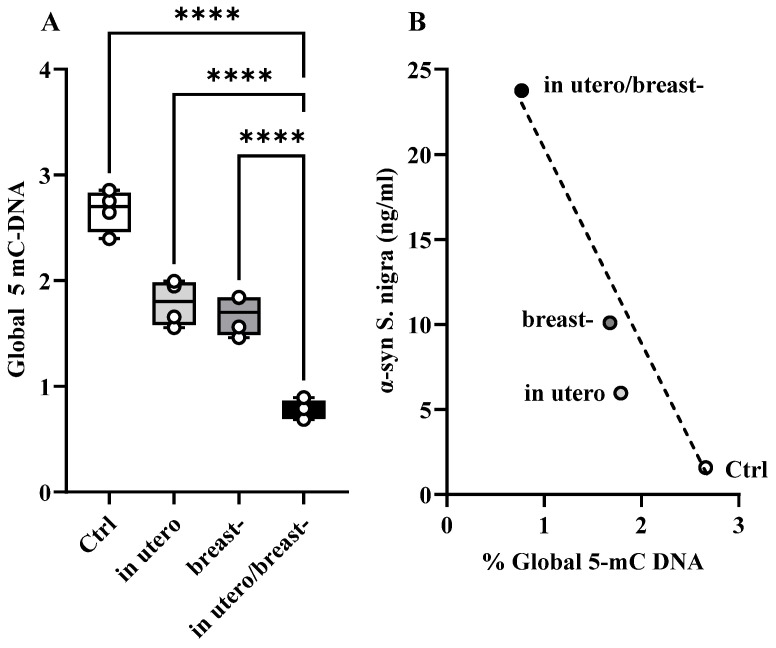
Graphs depict % Global 5-mC-DNA and its correlation with α-syn_if_. (**A**) Global %5-mC DNA (*n* = 4 for each group) and (**B**) Pearson’s linear negative correlation between α-syn_if_ and 5-mC DNA in *S. nigra*. Statistical analysis indicated a direct proportional relationship between the increase in α-syn_if_ content and the decrease in global 5-mC DNA levels. **** *p* < 0.001.

At 60 PND, offspring rats exposed to ROT during development exhibited increments in the α-syn_if_ levels in the *S. nigra* [Figure 4F], while global hypomethylation of 5-mC DNA (Figure 7A) was notoriously lower in the utero/breast group than in the Ctrl, whereas the breast and in utero were very similar, and corresponded to 0.7699, 1.789, 1.6676, and 2.662%, respectively. One-way ANOVA and Tukey’s multiple comparisons post hoc test showed that [F_(3,12)_ = 73.03, *p* < 0.0001; r^2^ = 0.948; Figure 7A]. Pearson’s linear correlation tests demonstrated a negative correlation between the concentration of α-syn_if_ and hypomethylation of 5-mC DNA in *S. nigra* [r = −0.95, *p* = 0.0439; Figure 7B].

## 4. Discussion

Traditionally, PD has been associated with aging and, in some cases, is linked to inheritable genes. However, updated perspectives on the etiology of this disease suggest that adult exposure to toxins such as agrochemicals can predispose organisms to PD later in life, indicating an environmental component. Recently, the concept that PD and other neurodegenerative diseases may have developmental origins has gained attention [39,40], although this has not been fully accepted.

Studies conducted by Greenamyre’s group at Emory University showed that systemic exposure of adult Lewis rats to ROT by the implantation of a subcutaneous osmotic pump at doses of 2–3 mg/kg/day induce highly selective nigrostratal dopaminergic lesions [27,28,29,30] and in our previous work [13], pregnant females were ROT-exposure at 0.2, 0.4, 0.6, and 1 mg/kg/day to determine the doses with higher survival of dams and offspring, and we demonstrated motor damage we demonstrated motor damage and progressive loss of nigrostriatal dopaminergic neurons in 30 and 60 PND in offspring Wistar rats probably by toxicity and apoptosis mediated by the increase of intracellular calcium and generation of reactive oxygen species (ROS), like found Swarnkar et al. (2012), in Neuro-2a cell ROT-exposed [41].

In this work, we strengthened this notion by providing evidence that ROT exposure at 1 mg/kg/día in utero and/or breast reduces the number of dopaminergic neurons in the *S. nigra* (Figure 4E), increases the availability of α-syn_if_ (Figure 4F,G), and impairs motor performance (Figure 3) when animals were evaluated at 60 days of age, despite the low circulating blood concentrations of ROT (Figure 5). However, it remains imperative to determine whether the loss of nigral dopaminergic neurons has an early onset, progresses gradually, or is abrupt, or whether it represents a latent stage prior to the development of the Parkinsonian phenotype. An important finding of our study was the differential expression of cytological, biochemical, and neurological alterations observed in the descendants of ROT-treated dams across the various groups. Specifically, the most significant alterations were observed in animals exposed to ROT in utero/breastfeeding, followed by significant effects in those exposed solely in utero and moderate effects in rats exposed only during breastfeeding. This work is strengthened by the findings of Ma et al. (2017), who exposed Sprague–Dawley rats at five gestation days until lactation to paraquat and maneb at doses of 15/45 mg/kg twice a week, and found that the combination of these pesticides damages dopaminergic neurons in the midbrain during development, due to an increase in the expression of Wnt5a and a decrease in the mRNA and protein levels of Wnt1, Nurr1, and TH; without modifying the concentration of dopamine in the striatum, neither differences between males and females exist in any of the parameters evaluated [42].

This underscores the fact that the impact of the toxicant varies depending on the developmental stage during which the exposure occurs. During both in utero and postnatal development of the nigrostriatal dopaminergic system, ROT may interact with and interfere with the proper development of progenitor stem cells and immature postmitotic neurons. Additionally, ROT may disrupt the activity of various transcription factors such as En-1, En-2, Nurr1, Lmx1B, and Pitx3, which play critical roles in neuronal migration, differentiation, maturation, and cell survival [43,44,45], thereby exerting neurotoxic effects. It is also likely that the hypomethylation breastfeeding ROT-exposure group were different to the Ctrl group, as we would expect, probably due to an individual condition of the breastfeeding group in which ROT by an unknown mechanism or neurotoxicity inhibits DNMT and causes the hypomethylation of 5mC-DNA. Hutnick et al. 2009 [46] found that the mutation of DNMT1 in mice impairs postnatal neuronal maturation of the cortex and hippocampus and showed neurobehavior defects in adulthood and neuronal death. Future studies are required to understand how specific changes in the epigenome in *S. nigra* and their correlation with other structures can induce neurodegeneration in Parkinson’s disease,

These results suggest the presence of sensitive periods, during which dopaminergic neurons may exhibit varying degrees of vulnerability to ROT exposure. Our results indicate that ROT exposure in utero appears to be more detrimental than exposure during breastfeeding, consistent with previous studies demonstrating ROT’s neurotoxic effects of ROT on adult nigral dopaminergic neurons [27,28,29,30]. The differences observed between the groups may be attributed to variations in the routes taken by ROT and its metabolites to reach the brain during the in utero stage (placental/hematogenic) versus the breastfeeding (intestinal/hematogenic–lymphatic) stage of life, as well as potential differences in the ability of each organism to detoxify ROT at different ages. Further studies aimed at assessing the long-term effects of ROT exposure during various stages of gestation, breastfeeding in adulthood, and old age, will offer more robust evidence to reinforce the concept of periods sensitive to environmental toxicants. Nonetheless, our results support the notion that adult-onset Parkinsonism may result from exposure to harmful environmental agents during prenatal and/or early postnatal life.

An interesting observation from our study was the increased availability of α-syn_if_ across all the experimental groups. Given that ROT inactivates proteasome activity [2,46], it is likely that the elevated presence of α-syn_if_ in ROT-exposed rats is due to reduced degradation of abnormal forms of this protein. Interestingly, the degree of increase in α-syn_if_ differed among the ROT-exposed groups. Despite the roughly equivalent duration of exposure to ROT in utero and in breastfeeding-exposed rats, this observation implies that the ubiquitin–proteasome system may be more sensitive to disruption of ROT during prenatal development. Moreover, there is evidence suggesting that ROT causes oxidative damage to α-syn [47]. Since the availability of α-syn_if_ increases during postnatal development [13], the increased accumulation of α-syn_if_ (Figure 4F,G) observed in our experimental rats might reflect the process of ROT-associated oxidative damage, considering that levels of α-syn tend to increase with age [48]. Additionally, hypomethylation of 5-mC DNA (Figure 6A) in CpG dinucleotides disrupted the transcriptional machinery of neurons. Matsumoto et al. [49] reported global hypomethylation in *S. nigra* in patients with PD, whereas Jowaed et al. [50] showed that hypomethylation of α-syn intron 1 leads to its overexpression, and Mpofana et al. [51] showed that exposure to early life stress results in epigenetic changes in a Parkinsonian rat model. This may explain our finding of a direct correlation between global DNA hypomethylation (% 5-mC DNA) and increased α-syn_if_ concentration in *S. nigra* (Figure 4F).

Environmental agents such as pesticides have the potential to inflict damage to both the developing and mature nervous systems, culminating in neurodegenerative diseases in adulthood. Epigenetic changes, which involve alterations in gene expression without modifications to the coding sequence, play pivotal roles in this process. These modifications include DNA methylation and histone modification. Early life exposure to ROT may induce the expression of neurodegenerative genes through epigenetic modifications, consequently contributing to late-onset neurodegenerative diseases. Research on the regulation of DNA methylation and histone modification may elucidate whether an individual’s genetic makeup is altered by environmental factors, thereby increasing the risk of neurodegeneration [52,53,54].

## 5. Conclusions

The three observations in our study are particularly puzzling. First, we did not observe consistent additive effects when the rats were exposed to ROT in utero or breastfeeding. Second, prolonged ROT exposure resulted in the elimination of 12% more TH-IR neurons in the *S. nigra* than did prenatal ROT exposure. While the implications of these findings remain unclear, we speculate that they may reflect (1) variations in the mechanism by which ROT enters the brain and undergoes metabolism at different developmental stages, (2) a potential difference in the susceptibility of early- versus late-generated neuroblasts to the drug, and/or (3) distinct vulnerability levels of maturing dopaminergic neurons. The third aspect, somewhat counterintuitive, is that in humans, a loss of more than 75% of dopaminergic neurons is needed to develop Parkinsonism [1]. However, in our experiments, motor impairments were observed, with only a 50% loss of TH-IR neurons in *S. nigra*. These observations suggest that, during neurodevelopment, rats may be more sensitive to the loss of dopaminergic neurons. However, these findings warrant further investigation.

Furthermore, our results have implications in humans. Indeed, a study conducted on pregnant and non-pregnant Mexican American women found higher levels of various pesticides, including ROT, in the blood samples of pregnant women than in non-pregnant women [55]. Similarly, it is worth questioning whether ROT exposure to rats has analogous effects on the nigral dopaminergic neurons in humans. It is plausible that exposure to ROT during pregnancy may produce offspring prone to developing Parkinsonism in adulthood compared to the offspring of women who are not exposed to ROT [55]. Based on our experimental observations, future epidemiological and basic research should be conducted to develop preventive measures.

## Data Availability

The raw data supporting the conclusions of this study are made available by the authors upon request.

## Supplementary Materials

The following supporting information can be downloaded at: https://www.mdpi.com/article/10.3390/toxics13040290/s1, Table S1. Offspring group Ctrl and ROT-exposure time, number of the litter by sex and survival percentage of offspring at 60 PND. Figure S1. Standard curve using linear regression α-synif (pg/mL). Figure S2. Standard curve using linear regression of total 5-mC DNA (%). Table S2. Average RGS in pregnant dams.

## References

[1] Fearnley J.M., Lees A.J. (1991). Ageing and Parkinson’s disease: *Substantia nigra* regional selectivity. Brain.

[2] McNaught K.S.P., Olanow C.W., Halliwell B., Isacson O., Jenner P. (2001). Failure of the ubiquitin-proteasome system in Parkinson’s disease. Nat. Rev. Neurosci..

[3] Feng Y., Liang Z.H., Wang T., Qiao X., Liu H.J., Sun S.G. (2006). Alpha-Synuclein redistributed and aggregated in rotenone-induced Parkinson’s disease rats. Neurosci. Bull..

[4] Williams-Gray C.H., Foltynie T., Lewis S.J.G., A Barker R. (2006). Cognitive deficits and psychosis in Parkinson’s disease: A review of pathophysiology and therapeutic options. CNS Drugs.

[5] Lonskaya I., Hebron M., Algarzae N., Desforges N., Moussa C.H. (2013). Decreased parkin solubility is associated with impairment of autophagy in the nigrostriatum of sporadic Parkinson’s disease. Neuroscience.

[6] Hindle J.V. (2010). Ageing, neurodegeneration and Parkinson’s disease. Age Ageing.

[7] Houlden H., Singleton A.B. (2012). The genetics and neuropathology of Parkinson’s disease. Acta Neuropathol..

[8] Puschmann A. (2013). Monogenic Parkinson’s disease and parkinsonism: Clinical phenotypes and frequencies of known mutations. Park. Relat. Disord..

[9] Gorell J.M., Rybicki B.A., Johnson C.C., Peterson E.L. (1999). Occupational metal exposures and the risk of Parkinson’s disease. Neuroepidemiology.

[10] Moretto A., Colosio C. (2011). Biochemical and toxicological evidence of neurological effects of pesticides: The example of Parkinson’s disease. Neurotoxicology.

[11] Cereda E., Cilia R., Klersy C., Siri C., Pozzi B., Reali E., Colombo A., Zecchinelli A.L., Mariani C.B., Tesei S. (2016). Dementia in Parkinson’s disease: Is male gender a risk factor?. Park. Relat. Disord..

[12] Cory-Slechta D.A., Thiruchelvam M., Richfield E.K., Barlow B.K., Brooks A.I. (2005). Developmental pesticide exposures and the Parkinson’s disease phenotype. Birth Defects Res. Part A Clin. Mol. Teratol..

[13] Gómez-Chavarín M., Diaz-Perez R., Morales-Espinosa R., Fernandez-Ruiz J., Roldan-Roldan R., Torner C. (2013). Developmental effects of rotenone pesticide exposure on the rat nigrostriatal dopaminergic system. Salud Ment..

[14] Perera F., Rauh V., Whyatt R., Tang D., Tsai W., Bernert J., Tu Y., Andrews H., Barr D., Camann D. (2005). A summary of recent findings on birth outcomes and developmental effects of prenatal ETS, PAH, and pesticide exposures. Neurotoxicology.

[15] Barlow B.K., Coryslechta D., Richfield E.K., Thiruchelvam M. (2007). The gestational environment and Parkinson’s disease: Evidence for neurodevelopmental origins of a neurodegenerative disorder. Reprod. Toxicol..

[16] Fleming S.M. (2017). Mechanisms of Gene-Environment Interactions in Parkinson’s Disease. Curr. Environ. Health Rep..

[17] Shan L., Heusinkveld H.J., Paul K.C., Hughes S., Darweesh S.K.L., Bloem B.R., Homberg J.R. (2023). Towards improved screening of toxins for Parkinson’s risk. NPJ Park. Dis..

[18] Luo W., Ruan D., Yan C., Yin S., Chen J. (2012). Effects of chronic lead exposure on functions of nervous system in Chinese children and developmental rats. Neurotoxicology.

[19] Bjorklund G., Stejskal V., Urbina M.A., Dadar M., Chirumbolo S., Mutter J. (2018). Metals and Parkinson’s Disease: Mechanisms and Biochemical Processes. Curr. Med. Chem..

[20] Vellingiri B., Suriyanarayanan A., Abraham K.S., Venkatesan D., Iyer M., Raj N., Gopalakrishnan A.V. (2022). Influence of heavy metals in Parkinson’s disease: An overview. J. Neurol..

[21] Mursaleen L.R., Stamford J.A. (2016). Drugs of abuse and Parkinson’s disease. Prog. Neuropsychopharmacol. Biol. Psychiatry.

[22] Hagberg H., Gressens P., Mallard C. (2012). Inflammation during fetal and neonatal life: Implications for neurologic and neuropsychiatric disease in children and adults. Ann. Neurol..

[23] Iacono S., Schirò G., Davì C., Mastrilli S., Abbott M., Guajana F., Arnao V., Aridon P., Ragonese P., Gagliardo C. (2023). COVID-19 and neurological disorders: What might connect Parkinson’s disease to SARS-CoV-2 infection. Front. Neurol..

[24] Channer B., Matt S.M., Nickoloff-Bybel E.A., Pappa V., Agarwal Y., Wickman J., Gaskill P.J. (2023). Dopamine, Immunity, and Disease. Pharmacol. Rev..

[25] Casper R.C. (2004). Nutrients, neurodevelopment, and mood. Curr. Psychiatry Rep..

[26] Valleau J.C., Sullivan E.L. (2014). The impact of leptin on perinatal development and psychopathology. J. Chem. Neuroanat..

[27] Betarbet R., Sherer T.B., MacKenzie G., Garcia-Osuna M., Panov A.V., Greenamyre J.T. (2000). Chronic systemic pesticide exposure reproduces features of Parkinson’s disease. Nat. Neurosci..

[28] Betarbet R., Sherer T.B., Greenamyre J.T. (2002). Animal models of Parkinson’s disease. Bioessays.

[29] Sherer T.B., Kim J.-H., Betarbeta R., Greenamyrea J.T. (2003). Subcutaneous rotenone exposure causes highly selective dopaminergic degeneration and alpha-synuclein aggregation. Exp. Neurol..

[30] Schmidt W.J., Alam M. (2006). Controversies on new animal models of Parkinson’s disease pro and con: The rotenone model of Parkinson’s disease (PD). J. Neural Transm. Suppl..

[31] Cannon J.R., Tapias V., Na H.M., Honick A.S., Drolet R.E., Greenamyre J.T. (2009). A highly reproducible rotenone model of Parkinson’s disease. Neurobiol. Dis..

[32] Ryan S.D., Dolatabadi N., Chan S.F., Zhang X., Akhtar M.W., Parker J., Soldner F., Sunico C.R., Nagar S., Talantova M. (2013). Isogenic human iPSC Parkinson’s model shows nitrosative stress-induced dysfunction in MEF2-PGC1α transcription. Cell.

[33] Neely M.D., Davison C.A., Aschner M., Bowman A.B. (2017). From the Cover: Manganese and Rotenone-Induced Oxidative Stress Signatures Differ in iPSC-Derived Human Dopamine Neurons. Toxicol. Sci..

[34] McKnight S., Hack N. (2020). Toxin-Induced Parkinsonism. Neurol. Clin..

[35] (1992). Mexican Official Standard Norm for Animal Care. Diario Oficial, México. https://www.gob.mx/cms/uploads/attachment/file/203498/NOM-062-ZOO-1999_220801.pdf.

[36] Drucker-Colín R., García-Hernández F. (1991). A new motor test sensitive to aging and dopaminergic function. J. Neurosci. Methods.

[37] Campbell B.C., Li Q.X., Culvenor J.G., Jäkälä P., Cappai R., Beyreuther K., Masters C.L., McLean C.A. (2000). Accumulation of insoluble alpha-synuclein in dementia with Lewy bodies. Neurobiol. Dis..

[38] Juraska J.M., Wilson C.J., Groves P.M. (1977). The substantia nigra of the rat: A Golgi study. J. Comp. Neurol..

[39] Landrigan P.J., Sonawane B., Butler R.N., Trasande L., Callan R., Droller D. (2005). Early environmental origins of neurodegenerative disease in later life. Environ. Health Perspect..

[40] Wexler E.M., Geschwind D.H. (2007). Out FOXing Parkinson disease: Where development meets neurodegeneration. PLoS Biol..

[41] Swarnkar S., Goswami P., Kamat P.K., Gupta S., Patro I.K., Singh S., Nath C. (2012). Rotenone-induced apoptosis and role of calcium: A study on Neuro-2a cells. Arch. Toxicol..

[42] Ma J., Huang C., Ma K., Wu Y.P., Li B.X., Sun Y. (2017). Effect of Wnt1 and Wnt5a on the development of dopaminergic neurons, and toxicity induced by combined exposure to paraquat and maneb during gestation and lactation. Mol. Med. Rep..

[43] Vitalis T., Cases O., Parnavelas J.G. (2005). Development of the dopaminergic neurons in the rodent brainstem. Exp. Neurol..

[44] Ang S.L. (2006). Transcriptional control of midbrain dopaminergic neuron development. Development.

[45] Alavian K.N., Scholz C., Simon H.H. (2008). Transcriptional regulation of mesencephalic dopaminergic neurons: The full circle of life and death. Mov. Disord..

[46] Betarbet R., Canet-Aviles R.M., Sherer T.B., Mastroberardino P.G., McLendon C., Kim J.-H., Lund S., Na H.-M., Taylor G., Bence N.F. (2006). Intersecting pathways to neurodegeneration in Parkinson’s disease: Effects of the pesticide rotenone on DJ-1, alpha-synuclein, and the ubiquitin-proteasome system. Neurobiol. Dis..

[47] Mirzaei H., Schieler J.L., Rochet J.-C., Regnier F. (2006). Identification of rotenone-induced modifications in alpha-synuclein using affinity pull-down and tandem mass spectrometry. Anal. Chem..

[48] Chu Y., Kordower J.H. (2007). Age-associated increases of alpha-synuclein in monkeys and humans are associated with nigrostriatal dopamine depletion: Is this the target for Parkinson’s disease?. Neurobiol. Dis..

[49] Matsumoto L., Takuma H., Tamaoka A., Kurisaki H., Date H., Tsuji S., Iwata A. (2010). CpG demethylation enhances alpha-synuclein expression and affects the pathogenesis of Parkinson’s disease. PLoS ONE..

[50] Jowaed A., Schmitt I., Kaut O., Wüllner U. (2010). Methylation regulates alpha-synuclein expression and is decreased in Parkinson’s disease patients’ brains. J. Neurosci..

[51] Mpofana T., Daniels W.M.U., Mabandla M.V. (2016). Exposure to Early Life Stress Results in Epigenetic Changes in Neurotrophic Factor Gene Expression in a Parkinsonian Rat Model. Park. Dis..

[52] Mattson M.P. (2003). Methylation and acetylation in nervous system development and neurodegenerative disorders. Ageing Res. Rev..

[53] Kwok J.B. (2010). Role of epigenetics in Alzheimer’s and Parkinson’s disease. Epigenomics.

[54] Wang Y., Wang X., Li R., Yang Z., Wang Y., Gong X., Wang X. (2013). A DNA methyltransferase inhibitor, 5-aza-2′-deoxycytidine, exacerbates neurotoxicity and upregulates Parkinson’s disease-related genes in dopaminergic neurons. CNS Neurosci. Ther..

[55] Woodruff T.J., Zota A.R., Schwartz J.M. (2011). Environmental chemicals in pregnant women in the United States: NHANES 2003–2004. Environ. Health Perspect..

