# Supplementary material for: Rotenone Exposure During Development Conditions Parkinsonian Phenotype in Young Adult Rats
Source: Toxics. 2025 Apr 10;13(4):290. doi: 10.3390/toxics13040290 (PMC12030936; doi:10.3390/toxics13040290)
Supplement: Supplementary file 1 [file toxics-13-00290-s001.zip › toxics-3506438-supplementary.pdf]

Supplementary Materials S1

Table S1. Offspring group Ctrl and ROT-exposure time, number of the litter by sex and survival percentage of offspring at 60 PND.

ROT-exposure/days	Offspring group	Male (number)	Female (number	% Survival 1 DPN	%Survival 60 DPN
0	Ctrl	10	8	100	100
21 (in gestation	in utero	8	10	100	75
21 (in breastfeeding)	breast	9	9	100	95
42 (in gestation/breast	in utero/breast	11	7	100	78

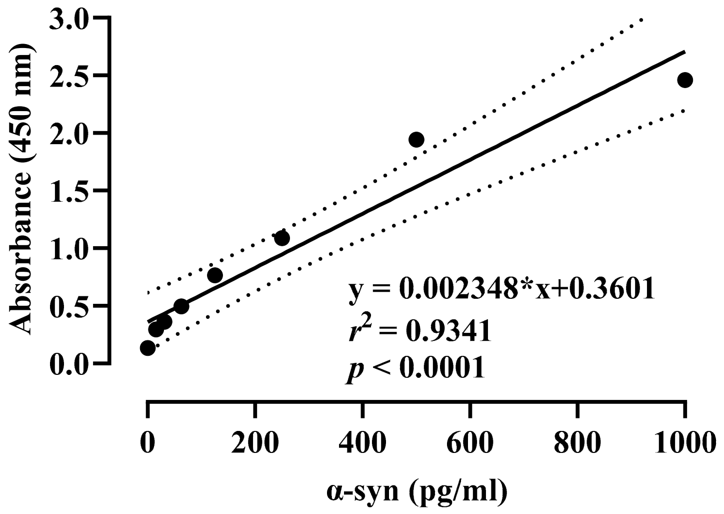

Figure S1. Standard curve using linear regression α-syn$_{lf}$ (pg/ml).

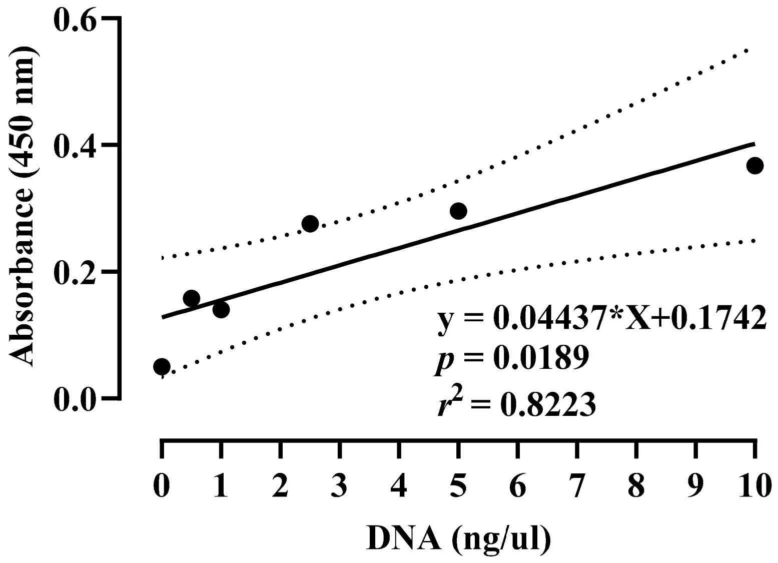

Figure S2. Standard curve using linear regression of total 5-mC DNA (%).

Supplementary Materials S2

Protocol for the care of animal welfare

In 2002, Betarbet et al. demonstrated that a dose of 12 mg/kg/day of ROT s.c. in Sprague–Dawley and Lewis rats reproduced the pathological characteristics of Parkinson's disease. The rats were euthanized between 5 and 35 days. Our study used 8 female Wistar rats: 4 control (untreated) and 4 were administered s.c. with 1 mg/kg/day of ROT for 42 days, corresponding to 21 days of gestation and 21 days of lactation. This dose was determined after performing a dose/response curve of: 0.2, 0.4, 0.6, and 1 mg/kg/day (Gómez-Chavarín, et al. 2013), with the latter being the dose that achieved the highest survival rate and determination of the number of subjects to use.

After the daily administration of 1 mg/kg/day of ROT, the subjects were observed on two occasions: minutes after administration and eight hours later. Additionally, they were evaluated weekly with the Rat Grimace Scale (RGS) and the animal welfare parameters considered in the protocols: 1) good feeding, 2)

good housing, 3) good health, and 4) ability to display species-specific behaviors, to assess the quality of life of the females exposed to ROT.

The RGS has been validated in various experimental rodent models and allows visual assessment of pain and discomfort and indirectly the physiological state through facial expressions in rodents. It includes five parameters: orbital tightening (OT), nose flattening (NF), cheek flattening (ChF), ear changes (ECh), and whisker changes (WCh), with a scale of 0 to 4, in addition to bristly hair. In no case did we find values of 4. The following Table 1 shows that the control females in the third week of gestation had an average RGS value of 0.5 to 1.0, indicative of discomfort close to childbirth. These same values are found in the first and second weeks of gestation in females exposed to ROT, increasing from 0.75 to 1.60 in the third week. These values increase due to the proximity of childbirth and the treatment; however, this did not prevent the birth of the offspring, which did not show birth defects.

Table S2. Average RGS in pregnant dams.

Group	1st week	2° week	3° week
Ctrl	0.00	0.00	0.5-1.00
ROT	0.00	0.75	1.3-1.60

Females exposed to ROT did not exhibit accelerated breathing, lack of mobility, or weight loss greater than 15% due to inability to eat or drink, conditions that would warrant euthanasia for humane reasons, which was not necessary at any time.

To estimate the number of offspring necessary for the protocols, it was calculated with n(1/1-R), where n represents the total number of offspring and R is the expected loss proportion of subjects, adding 10% for subjects exposed to ROT.

The loss of offspring was greater during the first three days after birth, mainly those exposed to ROT in utero and in utero/breast, corresponding to 25% and 22%, respectively (Supplementary Table 1), while those exposed only during

lactation had a loss of 5%. Offspring from groups exposed to ROT lost weight (Manuscript Figure 2), but there were no differences in the number of offspring per litter and the number/sex (Supplementary Table 1).

No behavioral or birth defects were observed in the offspring at birth, but motor deficiencies were observed when performing specific tasks such as the inclined beam, as shown in Figure 3 of the manuscript.
